# Supplementary material for: Assessing geographical variation in ovulatory cycle knowledge among women of reproductive age in Sierra Leone: Analysis of the 2019 Demographic and Health Survey
Source: PLoS One. 2024 Apr 16;19(4):e0300239. doi: 10.1371/journal.pone.0300239 (PMC11020968; doi:10.1371/journal.pone.0300239)
Supplement: S1 Checklist — (DOCX) [file pone.0300239.s001.docx]

***PLOS ONE* Clinical Studies Checklist**

***PLOS ONE* manuscript number:** PONE-D-23-11422

| **Complete the following if your study involved human participants or human subjects’ data. These questions should be addressed for prospective and retrospective studies.** | | |
| --- | --- | --- |
| 1. | Did you obtain ethics approval for this study?   - If yes, please upload (file type “Other”) the original approval document you received from your ethics committee. If the original document is in another language, please also provide an English translation.   ___ Uploaded ___ N/A   - If you did not obtain ethical approval, please explain why this was not required.  \| Our study reported on research involving human participants and as expected in such studies, an institutional review board (ethics committee) is required to review and approve the study before it began. In our specific study, however, ethical approval was not applicable since the DHS already had ethical clearance for conducting the primary survey in Sierra Leone and made the data available for general use by the public. Because we strictly followed the conditions of the institutional review board (ethics committee) that approved the DHS in Sierra Leone, we did not seek additional ethical approval in this specific case since our methodology was not intrusive on participants. Additionally, our methodology did not require informed consent from participants because we used secondary data (DHS from Sierra Leone). We, however, did request permission and written informed consent from the DHS program to use the DHS dataset which was approved before the study began. \| \| --- \| |  |
| 2. | If your study involved human participants, please report in the Methods section when participants were recruited to the study.  ___ Completed ___ N/A |  |
| 3. | If you are reporting a study of medical records or archived samples, please report in the Methods section the date range in which human subjects’ data/samples were collected and the date(s) when you conducted this study.  ___ Completed ___ N/A |  |
| 4. | Please specify in the Methods section whether authors had access to information that could identify individual participants during or after data collection.  ___ Completed ___ N/A |  |
| 5. | If you are reporting an observational study – i.e. cohort, case-control, and cross-sectional studies – we recommend that the work is reported as per the requirements of the STROBE guidelines, and that you provide a completed STROBE checklist as a Supporting Information file with your submission.  The STROBE checklist was developed to improve the reporting of observational human subjects research, and is available here: <http://strobe-statement.org/fileadmin/Strobe/uploads/checklists/STROBE_checklist_v4_combined_PlosMedicine.docx>.  ___ Completed ___ N/A |  |
| 6. | Please ensure that the author list and Corresponding Author entered in Editorial Manager match the author list and Corresponding Author in your manuscript file.  ___ Completed |  |
