# Supplementary material for: Assessing geographical variation in ovulatory cycle knowledge among women of reproductive age in Sierra Leone: Analysis of the 2019 Demographic and Health Survey
Source: PLoS One. 2024 Apr 16;19(4):e0300239. doi: 10.1371/journal.pone.0300239 (PMC11020968; doi:10.1371/journal.pone.0300239)
Supplement: Attachment — Submitted filename: PLoS One_Review_Ovulation Knowledge.docx [file pone.0300239.s002.docx]

This is an interesting project that explores an important area of sexual and reproductive health. The authors have done a great job but a few of these suggestions can make the work better.

**Abstract**

*“We investigated regions of Sierra Leone and other sociodemographic variables to determine where the needs for improved ovulatory cycle knowledge are greatest”.*

This appears unclear. “Investigated regions” makes the sentence confusing. Maybe it can be written as: **We investigated regions of Sierra Leone where the need for improved ovulatory cycle knowledge is greatest and associated sociodemographic variables.**

Conclusion of the abstract: I think “understanding” should be added to the sentence that states: …that stakeholders should integrate comprehensive education on the importance of **UNDERSTANDING** the ovulatory cycle and its timing, into targeted family planning and reproductive health educational program

**Background**

Great job on the well-written background section, albeit I have a few comments.

- Indicators of ovulation are not exhaustive, because there might be other signs of ovulation that other women experience that have not been added here, I think the authors should make this obvious by adding “etc”.
- Has there been any similar study on this topic in the context of Sierra Leone? The authors appear very silent on this, if not, aside from informing programs and interventions, I think the authors should state that their study will fill this gap in knowledge.

**Method**

- Can a weblink for the DHS data be included somewhere in the Data Sources?
- In the data source, the authors should provide information about the data collection agency at the national level in Sierra Leone and any other international or local agencies that provided technical and financial assistance.
- Was there any rationale for not merging the exposure to mass media measured as the frequency of reading newspapers/magazines, listening to the radio, or watching TV? It is common in most studies to see a single measure of exposure to mass media based on these variables.
- Were there missing values in the data? How were they treated in the data analysis?

**Results**

- In the interpretation of the binary logit regression results, the authors should be careful of anthropomorphism. Hence, I feel the result should read “Women from the Northwestern region compared to the reference group of the Northern region” and other narratives should be directed to the women.

**Discussion**

- The study limitation/weakness stated by the authors appears too generic, and I do not see any reason why a longitudinal relationship between the region of residence/SES/demographic factors and the outcome interest would be needed.
