# Supplementary material for: Assessing geographical variation in ovulatory cycle knowledge among women of reproductive age in Sierra Leone: Analysis of the 2019 Demographic and Health Survey
Source: PLoS One. 2024 Apr 16;19(4):e0300239. doi: 10.1371/journal.pone.0300239 (PMC11020968; doi:10.1371/journal.pone.0300239)
Supplement: Attachment — Submitted filename: Response to Reviewers Comments-1_.docx [file pone.0300239.s003.docx]

**RESPONSE TO REVIEWERS’ COMMENTS.**

**REVIEWER #1:**

This is an interesting project that explores an important area of sexual and reproductive health. The authors have done a great job but a few of these suggestions can make the work better.

**Abstract**
“We investigated regions of Sierra Leone and other sociodemographic variables to determine where the needs for improved ovulatory cycle knowledge are greatest”. This appears unclear. “Investigated regions” makes the sentence confusing. Maybe it can be written as: We investigated regions of Sierra Leone where the need for improved ovulatory cycle knowledge is greatest and associated sociodemographic variables.

Response: Thank you, we have revised the statement and now reads “We investigated geographic region of Sierra Leone to determine where the needs for improved ovulatory cycle knowledge are greatest.”

**Conclusion of the abstract:** I think “understanding” should be added to the sentence that states: …that stakeholders should integrate comprehensive education on the importance of UNDERSTANDING the ovulatory cycle and its timing, into targeted family planning and reproductive health educational program

Response: This is done and now reads “Stakeholders should integrate education on the importance of understanding the timing of the ovulatory cycle into targeted family planning outreach programs.”

**Background**
Great job on the well-written background section, albeit I have a few comments.
o Indicators of ovulation are not exhaustive, because there might be other signs of ovulation that other women experience that have not been added here, I think the authors should make this obvious by adding “etc”.

Response: This is done.

o Has there been any similar study on this topic in the context of Sierra Leone? The authors appear very silent on this, if not, aside from informing programs and interventions, I think the authors should state that their study will fill this gap in knowledge.

Response: This is done, thank you.

**Method**
o Can a weblink for the DHS data be included somewhere in the Data Sources?

Response: A web link is added now, under “data source”.

o In the data source, the authors should provide information about the data collection agency at the national level in Sierra Leone and any other international or local agencies that provided technical and financial assistance.

Response: The partner organisations have been acknowledged.

o Was there any rationale for not merging the exposure to mass media measured as the frequency of reading newspapers/magazines, listening to the radio, or watching TV? It is common in most studies to see a single measure of exposure to mass media based on these variables.

Response: We were interested in how each of these media options relates with ovulatory cycle knowledge independently. Hence, merging them was going to defeat what we wanted to achieve.

o Were there missing values in the data? How were they treated in the data analysis?
Response: Yes, there were missing values in the data. However it was less than 5%, thus 1.8%, so they were dropped.

**Results**
o In the interpretation of the binary logit regression results, the authors should be careful of anthropomorphism. Hence, I feel the result should read “Women from the Northwestern region compared to the reference group of the Northern region” and other narratives should be directed to the women.

Response: We have made this revision.

**Discussion**
o The study limitation/weakness stated by the authors appears too generic, and I do not see any reason why a longitudinal relationship between the region of residence/SES/demographic factors and the outcome interest would be needed.

Response: We have modified this section.

**REVIEWER #2:**

This is an interesting piece that interrogate the geographical variable of level of ovulatory knowledge in Sierra Leone. Please find below a few observations for your attention
1. Arrange the key words alphabetically

Response: This is done.

2. Introduction
A. Kindly state the rationale for this study

Response: This is stated.

B. What is the gap that necessitates this study

Response: The association between region and ovulatory cycle knowledge has not been investigated in Sierra Leone and this is warranted since ovulatory cycle knowledge allows a woman to better plan her pregnancies or pregnancy avoidance. This gap has been written in the background section.

C. Is the issue peculiar to Sierra Leone and SSA?

Response: Yes, this is described in the introduction.

D. Why was the study conducted in Sierra Leone and not Ghana or Ethiopia?
Response: Ghana and Ethiopia's DHS datasets are older relative to Sierra Leone. Besides, Sierra Leone’s maternal health situation is more critical, as its maternal mortality ratio surpasses that of Ghana and Ethiopia. The background actually provides a justification on why this study was conducted in Sierra Leone.

E. Kindly state the specific objectives

Response: We have stated this in the background section.

3. **Method:** show the site where the dataset was curated (under the data source)
What is the meaning of "2019 Sierra Leone survey was part of DHS version VII)
Response: We have shown the site where the dataset was curated and the meaning of the statement has been provided under the Data Source section.

4. **Results**
A. Describe statistics and methods used to examine the subgroups. State the level of interactions between regions

Response: The statistics and methods for examining the regional sub-groups have been described in the “Data Analysis” and Results section.

5. Discussion
A. State the policy implications for the study

Response: This is done.

B. State the contribution to knowledge derived from the results

Response: This is done.
