# Supplementary material for: Assessing geographical variation in ovulatory cycle knowledge among women of reproductive age in Sierra Leone: Analysis of the 2019 Demographic and Health Survey
Source: PLoS One. 2024 Apr 16;19(4):e0300239. doi: 10.1371/journal.pone.0300239 (PMC11020968; doi:10.1371/journal.pone.0300239)
Supplement: Attachment — Submitted filename: Response to Reviewers Comments-2_.docx [file pone.0300239.s004.docx]

**RESPONSE TO REVIEWERS’ COMMENTS.**

**Reviewer #2:**

The observed comments have been included to enrich the quality of the manuscript. However, the authors have failed to state clearly the contribution this work has made to scholarly knowledge. Although, it has emphasized that lack of knowledge of the ovulatory cycle is a leading cause of unwanted pregnancy. Is this not a general knowledge? I will appreciate a clear cut contribution to scientific knowledge from this work, which is absolutely missing

**Response:**

Thank you for the feedback. However, we can't entirely agree with the reviewer's comments on this. We have provided some more specific clear cut contributions from this work to scientific knowledge as well as policy and programme decisions in Sierra Leone which have been highlighted in yellow for easy identification.
