# Supplementary material for: Assessing geographical variation in ovulatory cycle knowledge among women of reproductive age in Sierra Leone: Analysis of the 2019 Demographic and Health Survey
Source: PLoS One. 2024 Apr 16;19(4):e0300239. doi: 10.1371/journal.pone.0300239 (PMC11020968; doi:10.1371/journal.pone.0300239)
Supplement: Attachment — Submitted filename: Response to Reviewers Comments-5_.docx [file pone.0300239.s005.docx]

**RESPONSE TO REVIEWERS’ COMMENTS ON MANUSCRIPT TITLE: *ASSESSING THE GEOGRAPHICAL VARIATIONS IN OVULATORY CYCLE KNOWLEDGE AMONG WOMEN OF REPRODUCTIVE AGE IN SIERRA LEONE: ANALYSIS OF THE 2019 DEMOGRAPHIC AND HEALTH SURVEY*
 10/02/2024**

**Reviewer #2:**

This is a nice study that examined the link between ovulation and maternal mortality/NMR, and other pregnancy related complications.

**Response**
*Thank you*

It will be nice to add how many respondents were interviewed per household.

**Response**
*We did not capture the details about the demographics of respondents and the number interviewed per house in the manuscript since this information is already captured in the Sierra Leone Demographic and Health survey as reference [32] and may amount to duplication of information most especially since we used a secondary data. Nevertheless, out of the 13,399 households enlisted for the survey, data was collected from 15,574 respondents. Please see reference [32] for more detailed information as referenced.*

Also add the policy implications of this study.

***Response***

*This was captured in the conclusion statement of the abstract (‘This suggests that family planning outreach programs should include education on the ovulatory cycle and the importance of understanding the implications of its timing. This can reduce the risk of unintended pregnancies throughout Sierra Leone, but can have an especially positive impact in the Northwestern, Eastern, and Western regions, where ovulatory cycle knowledge was significantly lower’.)*

*Policy and programme implications were already indicated in the introduction statements of the main manuscript. But for emphasis the introduction statements of the manuscript was concluded to read: ‘All these have policy and programme implications to chart a path forward in the area of enhanced sexual and reproductive health of women in reproductive ages in Sierra Leone’.*

**Reviewer #4:**

**General comments:**
- Overall, the manuscripts lack an academic writing tone e.g. the manuscript is not attractive. The way the introduction of the manuscript was drafted is poor.

**Response**
*This is a very subjective statement which does to add any value to our manuscript. It must be indicated that the authors are seasoned academics with sound academic writing skills. We think the reviewer 4 should have been more constructive in the choice of words. However, reviewer 4 is entitled to his/her opinion.*

-The method should have been put in comparison to traditional methods or the advantage of the selected method over others, results should have clearly stated positive and negative associations separately, and conclusions should have more practical and actionable rather than showing directions. Thus, it would help if you redrafted the abstract.

**Response**
*The objective and focus of our study was not to compare ovulatory cycle knowledge as a family planning method with knowledge of other traditional family methods. These are two different study objectives which can be considered in a future study but for now that is not the focus of this current* *study.*

**Introduction**
- I don’t think you can discuss this issue like you presented in the introduction section.

**Response**

*We think this is the best way to this the issue based on our study objective*

- In the first place you need to define what ovulatory cycle knowledge is

**Response**

*The reviewer’s comments clearly show that he/she did not read the manuscript carefully. Most of the comments being made so far are not based on careful review of our manuscript. This particular comment about definition of ovulatory cycle knowledge has been well captured and explain in the introduction paragraph and with clear citations as follows: (‘Ovulation generally occurs at about the midpoint of the menstrual cycle. Therefore, for the average menstrual cycle of 28 days, ovulation will occur about 14 days after the onset of menstruation (i.e., when the period begins) [4]. It is important to note however that there can be variation in menstrual cycle length, which means that the occurrence of ovulation can vary from the typical 14-day mark [5]. In any case, menstruation can be used to estimate when one will ovulate [3]. Women who have a basic knowledge of the aforementioned physiological phenomena are better able to time their pregnancies, or avoid pregnancy if that is their goal [2,3]’).*

- For whom it is important

**Response**
*This has already been addressed in the manuscript. Please read the introduction and conclusion carefully.*

- I think you need a strong argument to show the topic is still valid in the presence of modern contraceptive methods.

**Response**
*I think the reviewer lacks in-depth knowledge about modern contraceptive methods particularly those that relate to natural family planning. In the light of current literature on contraception/family planning, the topic is still valid in the presence of modern contraceptive methods since not everybody is comfortable using ‘artificial’ contraceptive.*

This is more of a traditional method and we do not recommend people follow it because of the costly mistakes, and differences in women's body physiology as the symptoms and signs you mentioned can only occur in some women. I won’t say it is not searchable but you need to play in the context and give enough ground to prove it important.

**Response**
*We disagreed with this comment since literature has shown that despite the failure rate, it is very effective for those who know how to use this method correctly. Besides not every contraceptive has 100% success rate.*

- Starting from the abstract to the introduction you need to answer the why questions, where is the gap, do we need this information why, and where will this information be applicable?

**Response**
*This is out of place. This study is not an action research neither is it designed to answer questions on why, and where. It is purely a quantitative cross-sectional study that used secondary data from the 2019 Demographic and Health Survey (DHS) of Sierra Leone to examine a family planning situation.*

- Is this information important for policy decisions? Who is going to use this information why since modern contraception has better applicability?

**Response**
*Again, the reviewer should read the manuscript carefully. All these questions have already been addressed in details.*

- Thus, redraft the introduction section to fit your aim, and in this way, it will reveal what I can't see now from the existing context.
**Response**
*Again, the reviewer should read the manuscript carefully. All these questions have already been addressed in details.*

**Methods, results, and discussion**
- Say something about DHS. Which type of DHS is the data coming from is that mini or main?

**Response**
*Again, the reviewer should read the manuscript carefully. All these questions have already been addressed in details. The Reviewer obviously does not understand the meaning and rational, indications of Demographic and Health Surveys (DHS)*

- How data customized for analysis should be discussed

**Response**
*This is not relevant in this particular study methodology.*

- How missing data handled should be presented more clearly

**Response**
*This is an analysis of secondary data hence this comment is not relevant in this particular study methodology.*

- Results should be summarized in the same format and follow what was described in the method section

**Response**
*This is an analysis of secondary data hence this comment is not relevant in this particular study methodology.*

- Discussion should be formatted considering the contest requested above

**Response**
*This is an analysis of secondary data hence this comment is not relevant in this particular study methodology.*
